# Supplementary material for: The Effects of Fermentation of Low or High Tannin Fava Bean-Based Diets on Glucose Response, Cardiovascular Function, and Fecal Bile Acid Excretion during a 28-Day Feeding Period in Dogs: Comparison with Commercial Diets with Normal vs. High Protein
Source: Metabolites. 2021 Dec 16;11(12):878. doi: 10.3390/metabo11120878 (PMC8705793; doi:10.3390/metabo11120878)
Supplement: Supplementary file 1 [file metabolites-11-00878-s001.zip › metabolites-1434447-supplementary.pdf]

Supplementary Material

Supplementary Table S1. Diet formulation for lab-formulated fava bean diets (%).

Ingredient	Unfermented		Fermented	
	Low tannin	High tannin	Low tannin	High tannin
Wheat flour	53.28	53.20	53.28	53.20
Low tannin fava bean fermented	-	-	30.00	-
Low tannin fava bean unfermented	30.00	-	-	-
High tannin fava bean fermented	-	-	-	30.00
High tannin fava bean unfermented	-	30.00	-	-
Turkey meal	11.74	13.04	11.74	13.04
Canola oil	1.40	1.00	1.40	1.00
Celite^{TM}	1.00	1.00	1.00	1.00
Vitamin mixture	1.00	1.00	1.00	1.00
Mineral mixture	0.10	0.10	0.10	0.10
Salt	0.30	0.30	0.30	0.30
Dicalcium phosphate	0.53	0.26	0.53	0.26
Calcium carbonate	0.55	-	0.55	-
Choline chloride	0.10	0.10	0.10	0.10

Supplementary Table S2. Ingredient composition of normal and high protein commercial diets. Ingredients are listed in order of decreasing inclusion.

**Normal protein commercial diet**
Chicken, Brewers Rice, Whole Grain Wheat, Poultry By-Product Meal (Natural Source of Glucosamine), Corn Gluten Meal, Whole Grain Corn, Animal Fat Preserved with Mixed-Tocopherols (Form of Vitamin E), Corn Germ Meal, Fish Meal (Natural Source of Glucosamine), Animal Digest, Dried Egg Product, Salt, Potassium Chloride, Calcium Phosphate, Calcium Carbonate, Vitamin E Supplement, Choline Chloride, Zinc Sulfate, Ferrous Sulfate, L-Ascorbyl-2-Polyphosphate (Source of Vitamin C), L-Lysine Monohydrochloride, Manganese Sulfate, Niacin, Vitamin A Supplement, Calcium Pantothenate, Thiamine Mononitrate, Copper Sulfate, Riboflavin Supplement, Vitamin B-12 Supplement, Pyridoxine Hydrochloride, Garlic Oil, Folic Acid, Vitamin D-3 Supplement, Calcium Iodate, Biotin, Menadione Sodium Bisulfite Complex (Source of Vitamin K Activity), Sodium Selenite.
**High protein commercial diet**
Chicken Meal, Turkey Meal, Salmon Meal, De-Boned Chicken, De-Boned Turkey, De-Boned Trout, Potatoes, Chicken Fat (Preserved With Mixed Tocopherols), Peas, Tapioca, Lentils, Duck Meal, Chickpeas, Natural Chicken Flavor, Whole Dried Egg, Apples, Herring Meal, Flaxseed, Salmon Oil, Alfalfa, De-Boned Duck, De-Boned Salmon, Sweet Potatoes, Potassium Chloride, Pumpkin, Carrots, Bananas, Blueberries, Cranberries, Broccoli, Blackberries, Squash, Papayas, Pomegranate, Dried Chicory Root, Dried Lactobacillus Acidophilus Fermentation Product, Dried Enterococcus Faecium Fermentation Product, Dried Aspergillus Oryzae Fermentation Extract, Dried Bacillus Subtilis Fermentation Extract, Choline Chloride, Vitamins (Vitamin A Supplement, Vitamin D3 Supplement, Vitamin E Supplement, Niacin, L-Ascorbyl-2-Polyphosphate (A Source Of Vitamin C), D-Calcium Pantothenate, Thiamine Mononitrate, Beta-Carotene, Riboflavin, Pyridoxine Hydrochloride, Folic Acid, Biotin, Vitamin B12 Supplement), Minerals (Zinc Proteinate, Iron Proteinate, Copper Proteinate, Zinc Oxide, Manganese Proteinate, Copper Sulphate, Ferrous Sulphate, Calcium Iodate, Manganous Oxide, Selenium Yeast), Sodium Chloride, Taurine, Yucca Schidigera Extract, Dried Rosemary, Green Tea Extract, Peppermint, Parsley, Rosehips, Zedoary, Dandelion, Chamomile, Ginger, Fennel, Turmeric, Juniper Berries, Licorice, Marigold Extract, Cardamom, Cloves.

Supplementary Table S3. Proximate composition (%, dry matter basis) of lab-formulated fava bean diets compared to commercial diets.

**Item**	**Unfermented**		**Fermented**		**Commercial**	
	**Low tannin**	**High tannin**	**Low tannin**	**High tannin**	**Normal protein**	**High protein**
Moisture	9.53	9.11	7.80	9.10	6.69	5.84
Dry matter	90.47	90.89	92.20	90.90	93.31	94.16
Crude protein	27.42	27.25	27.94	27.98	31.25	40.73
Crude fiber	1.05	0.47	0.50	0.52	2.32	1.05
Fat	2.71	2.51	2.07	2.15	13.45	17.38
Ash	7.20	6.74	7.01	6.67	6.84	10.07
Cystine	0.35	0.63	0.48	0.57	0.63	0.15
Methionine	0.32	0.29	0.31	0.30	0.50	0.61
Taurine	0.06	0.06	0.06	0.06	0.09	0.23
Non-fiber carbohydrates	60.72	62.12	61.56	61.77	45.21	29.83
Total digestible nutrients	82.85	83.88	83.57	83.90	81.91	79.94
Metabolizable energy (kcal/g)	3.72	3.80	3.76	3.80	4.12	4.09
Insoluble HMWDF† % (w/w)	3.90	3.00	4.00	3.60	5.00	4.60
Soluble HMWDF† % (w/w)	0.70	0.90	0.70	0.90	0.50	1.40
Total HMWDF† % (w/w)	4.60	3.90	4.70	4.50	5.50	6.00
Vicine (mg/g)	1.76	1.77	0.36	0.61	-	-
Convicine (mg/g)	0.50	0.58	0.12	0.22	-	-

†High Molar Weight Dietary Fiber
